# Phototactic Behavior of the Armand Pine Bark Weevil, *Pissodes punctatus*

**DOI:** 10.1673/031.013.0301

**Published:** 2013-01-08

**Authors:** You Chen, Chang W. Luo, Rong P. Kuang, Hong W. Li, Zheng Chen, Ying J. Liu

**Affiliations:** 1 Life Science College, Yunnan University, Kunming, 650091 P. R. China; 2 Yunnan Forestry Technological College, Kunming 650224 P. R. China; 3 Research Institute of Resource Insects, Chinese Academy of Forestry Sciences, Kunming 650224 P. R. China; 4 Conservation Biology Department, Southwest Forestry University, Kunming 650224 P. R. China; 5 Insect Phototaxis Lab, Pesticide Eco-Alternatives Center, Kunming 650224 P. R. China

**Keywords:** light intensity, monochromatic light, ultraviolet light, violet light

## Abstract

The Armand pine bark weevil, *Pissodes punctatus* Langor et Zhang (Coleoptera: Curculionidae) is a destructive bark weevil on the Armand pine, *Pinus armandii* Franch (Pinales: Pinaceae), an important timbering tree in southern China. This study examined the phototactic behavior ï*éP. punctatus* through observation of behavioral characteristics, response to nine monochromatic lights (ranging from 340 nm to 689 nm with about 40-nm step), and response to five intensities (ranging from 1 lux to 200 lux) of the most attractive light. The results demonstrated that *P. punctatus* was most active in the day, and kept still at night (or in a dark room). *P. punctatus* could be attracted to eight of nine monochromatic lights, the exception being red light (649 nm), which implied broad sensitivity to the spectrum of light. *P. punctatus* was most sensitive to violet (415 nm), ultraviolet (340 nm), and green (504 nm) light, suggesting there might be at least three types of photoreceptors in the compound eyes of this weevil. Furthermore, low intensities elicited an increased phototactic response, and high intensities a decreased phototactic response, under both violet and UV light. Thus, *P. punctatus* were found to be phototactic insects, and the phototactic behavior of *P. punctatus* is both a color and intensity preference. The information provided here provides a basis for the improvement of trapping devices for detection and survey of *P. punctatus*, as well as a basis for the development of alternate control strategies for this important pest of Armand pine and other pine trees.

## Introduction

Phototaxis is a common behavior observed in many insects. Phototaxis can have several advantages for insects. These advantages include the finding of phototrophic organisms for food and the facilitation of the adult dispersal (Bakke 1968; Hamid 1995). Phototaxis is positive or negative movement along a light gradient. Insects can sense different spectrums of light, from the broad (Agee et al. 1990; Chen et al. 2009; Ju et al. 2010; Wei et al. 2000) to the narrow (Jaeger and Halman 1971). Many insects show a preference to shorter wavelengths such as UV, violet, and blue light (Yang et al. 2003; Guz et al. 2010; Fernando and Joseph 2011; Chen et al. 2012). In many insects, the sign of phototaxis depends on the intensity of light, so that low intensities elicit an increased response (Guo and Li 1997; Ye 2000), while increased intensities elicit a decreased response in some monochromatic lights (Chen et al. 2012). Thus, phototaxis of insects can be associated with both the spectrum and intensity of light, and can serve to bring insects towards the zones where they can potentially contact phototrophic organisms.

The Armand pine bark weevil, *Pissodes punctatus* Langor et Zhang (Coleoptera: Curculionidae), is a destructive stem-boring weevil that can feed on 17 pine tree species and can lay eggs on eleven of those pine trees in the south of China (Duan et al 1998). However, the most suitable host is Armand pine, *Pinus armandii* Franch (Pinales: Pinaceae), an important afforestation and timbering tree species in Southern China (Li et al. 2007). *P. punctatus* is difficult to control because of its hidden life, generation overlap, and long emergence period of adults (ranging from six to nine months in different places) (Duan et al 1998). Many studies suggested probable control measures of *P. punctatus*, including biological (Li et al. 2007; Yang et al. 2007), chemical (Liu et al. 2005), pherochemical (Wang et al. 2009), and ecological regularities (Li et al. 2000; Li et al. 2001). However, efficient, large-scale control measures are not provided at present (Chai and Liang 1990; Duan et al. 1998; Li et al. 2007).

Many previous studies indicated that the phototaxis of the beetle could be used for its control (Hamid et al. 1995; Ju et al. 2010; Fernando and Joseph 2011). For example, the damage of a different pine weevil, *Pissodes strobi* (Peck), was reduced by increasing canopy appropriately to intercept most of the ultraviolet light that strongly stimulates the weevils (see Hamid et al. 1995). Thus, phototactic behavior might be utilized for the control of phototactic weevils. For *P. punctatus*, previous studies reported some of its biological characteristics, including life history (Li et al. 2000) and its two ways of movement (flying and crawling); both ways of movement are hypothesized as mainly dispersal ways (Duan et al. 1998). However, all these observations were conducted only in the daytime. Thus, whether *P. punctatus* is a phototactic insect or not is still unknown.

Because *P. punctatus* disperses among different plantations of Armand pine quickly (Duan et al 1998), it was hypothesized that this weevil disperses mainly by flight and might be sensitive to light, which contributes significantly to its moving. In this paper, the following measurements of behaviors were examined: (1) the phototactic and dispersal behavior characteristics of *P. punctatus*, (2) the response to the spectrum of light using a bioassay tube, (3) the response to light intensity using a bioassay tube.

## Materials and Methods

### Study sites

The behavior characteristics of *P. punctatus* adults were observed at Zhehai wood farm (26° 25.547 N, 103° 35.536 E, 2670 m.a.s.l.) and at a laboratory (25° 05.882 N, 102° 46.317 E, 1985 m.a.s.l.) in Yunnan, China. Annual average temperature and precipitation at Zhehai wood farm is 11.4° C and 923.1 mm, respectively. Most of the precipitation occurs during the rainy season (from late May to late October). The canopy cover of Armand pine plantations ranged from 0.3 to 0.4. Both of the phototactic experiments were conducted for *P. punctatus* adults in Kunming, China.

### Behavior characteristics

To characterize the activity rhythm of *P. punctatus*, activity time and method of dispersal (crawling or flight) were observed. Only diurnal observation was conducted in Armand pine plantations in May 2011. Both diurnal and nocturnal observations were conducted in the laboratory in May 2012. For nocturnal observation in a dark room or at night, a flashlight covered with a red filter was used. Flight was observed on an Armand pine in an open yard at Zhehai wood farm in the daytime, and the flight distances of the weevil were recorded. Observations were repeated ten times.

### Insect collection

*P. punctatus* adults were collected from Armand pine at Zhehai wood farm in mid-May 2011 and mid-April 2012. The attacked host-trees were cut down, sawn into logs, sealed up at both ends with white latex, and brought back to the laboratory in Yunnan Forestry Technological College. These logs were then wrapped with plastic film and placed into four intelligent artificial climate incubators until the larvae and pupae of *P. punctatus* emerged into adults. The adults were collected and fed with the fresh branches of Armand pine in incubators. Only healthy and active adults were selected for phototactic response experiments. Before each phototactic experiment, the weevils were placed under indoor diurnal light for two hours and then placed in a dark room for one hour in order to adapt them to standard conditions.

**Figure 1.**
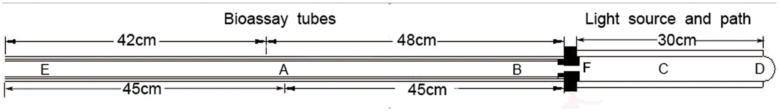
Device used for the phototactic experiments with *Pissodes punctatus* (A: Releasing point of the weevil; B and E: Measuring area of weevil displacement; C: Light source area; D: Tungsten halogen lamp; F: Silicone rubber joint). High quality figures are available online.

### Bioassay tube for phototactic experiment

Both of the phototactic experiments were conducted with the same bioassay tube in a dark room at 25 ± 1° C (Figure 1). The bioassay tube consisted of the following three parts:

(1) Light source and light path. A 50 W tungsten halogen lamp was used as light source. The halogen lamp (Figure 1D) was placed in a 30-cm-long steel tube (Figure 1C), which was connected to the end of the bioassay tube (Figure 1B) via a siliconerubber joint imbedded with an optical filter (Figure 1F). Light intensity was controlled with a slide rheostat and was measured with an illuminometer (Smart sensor AR823, http://smartsensor.en.alibaba.com/product/205 518253-200174142/Digital_Lux_Meter_AR823_1_200_000_LUX_.html) for violet light and with a UV light meter (UV513AB, General Tools, http://www.generaltools.com/) for UV light.

(2) Bioassay tube. The tube consisted of two pieces of inner tubes and two pieces of outer tubes (Figure 1B, E). Inner tubes were made of plexiglass (3.0 cm diameter), while outer tubes were made of polypropylene random copolymer with blackened surface to keep it light-tight (3.5 cm diameter). The lengths of both inner tubes were 45 cm, while the lengths of the two outer tubes were 48 cm and 42 cm. A black line was drawn on each of the two inner tubes at 10 cm from the releasing point (Figure 1A) in order to observe the position of the weevil.

(3) Optical filters, silicone-rubber joint. Optical filters (Huibo Optical Technology Co. Ltd., http://www.hboptical.com/CPJS/kejianzaidai.htmChina) with different center wavelengths of light were fixed in the center of a hollow, siliconerubber plug (Figure 1F). The silicone-rubber plug was polished first in order to keep the bioassay tube light-tight between the steel tube and bioassay tube.

### Trending response to monochromatic light

To test the phototactic behavior of *P. punctatus* to the spectrum of light, the response of the weevil to nine monochromatic lights was observed from late April to late May in 2012. An individual weevil was released from the middle of the inner tube (Figure 1A) and was permitted to crawl freely to each direction of the bioassay tube. Each treatment consisted of 10 weevils and was replicated five times. Eleven treatments (nine monochromatic lights and two controls) were assigned to 550 weevils (10 weevils/treatment × 11 treatments/replicate × 5 replicates). The following three experiments were performed:

(1) Total darkness. Both ends of the bioassay tube were opened in a dark room. This control assessed the behavior of the weevil without the stimulus of light and therefore allowed the evaluation of whether or not the displacement of the weevil was due to light stimuli.

 (2) Total light. Both ends of bioassay tube were opened to natural, diurnal light. This control assessed the behavior of the weevil under natural, diurnal light and therefore allowed the evaluation of whether or not the displacement of the weevil was due to the light.

 (3) Phototactic test. One end of bioassay tube (Figure 1E) was opened to darkness, while the other end of bioassay tube (Figure 1B) was opened to one monochromatic light. The intensity of the filtered light was always kept at 10 lux. Nine different optical filters were used, with center wavelengths of monochromatic light ranging from 340 nm to 689 nm with ∼40-nm step, including 340 nm, 381 nm, 415 nm, 451 nm, 504 nm, 549 nm, 601 nm, 649 nm, and 689 nm. This test assessed the phototactic behavior of the weevil under darkness and a specific wavelength of light and therefore allowed the assessment of which specific spectrum of light played an important role in the displacement of the weevil.

A weevil was considered to make a choice when it crossed the 10-cm black line drawn on the inner tubes. If it didn't cross the black line within 3 minutes of being released, it was considered to be non-responsive. In both the test of total darkness and the test of total light, all the weevils that crossed either of the two 10-cm black lines were recorded as being responsive. Three minutes was chosen as the time limit because the displacement of most weevils within 3 minutes was less than 43 cm in a test of the crawl speed of weevils in a bioassay tube under natural diurnal light. The trending rate was calculated as the number of responsive weevils to the number of total tested weevils. After 10 individuals had been tested, the inner tube was replaced with a clean one that had been washed with soapy water, rinsed with 95% alcohol, and dried with an electronic hair dryer. The position of the light source was changed as well. The plastic plug was changed when the treatment changed. Each weevil was used only once in order to avoid the influence of learned behavior.

### Response to light intensity

To test the response of *P. punctatus* to light intensity, the displacement of the weevil was recorded under different light intensities of violet light (415 nm) and UV light (340 nm) from mid-May to mid June 2012. This experiment shared the same device and the same method as the experiment of the response to monochromatic light. One end of the bioassay tube (Figure 1E) was opened to darkness, while the other end of the bioassay tube (Figure 1 B) was opened to eight intensities of violet (415 nm) and UV (340 nm) light, including 1 lux, 2 lux, 5 lux, 10 lux, 20 lux, 50 lux, 100 lux, and 200 lux. Treatment time was 3 minutes in each test. The displacement of each weevil from the point of release to its location after 3 minutes was recorded. In each treatment, ten weevils were used. Each treatment was replicated five times. 400 total weevils were tested (10 weevils/treatment × 8 treatments/replicate × 5 replicates). Each weevil was used only once in order to avoid the influence of learned behavior.

### Statistic analysis

The effect of the monochromatic light on trending rate and the effect of light intensity on displacement were analyzed by ANOVA and LSD. Both the trending rate and displacement data were checked for normal distribution and homoscadesticity and were found to meet the ANOVA requirements. All analyses were carried out with SPSS 11.5 (SPSS Inc., http://www-01.ibm.com/software/analytics/spss/). All means are shown with the standard error (± SE) of the mean throughout the text.

## Results

### Behavior characteristics

When being fed in incubators, *P. punctatus* were most active during the daytime and often kept still at night or in a dark room during the daytime. In natural plantations, *P. punctatus* were most active on sunny days, and preferred to feed on the base of leaf sheaths and on oneyear old shoots. On cloudy and rainy day, *P. punctatus* crawled along trunks and hid themselves under the bark of the host-trees. They usually stayed withing one tree (n = 32), but occasionally crawled to another tree (n = 1). If a weevil fell flat on its back, it could not turn itself over easily. Adults could fly away from resting needles when the needles were gently shaken during the daytime, flying an average distance of 42.5 ± 7.2 m (n = 10) per flight.

### Response to monochromatic light

**Figure 2.**
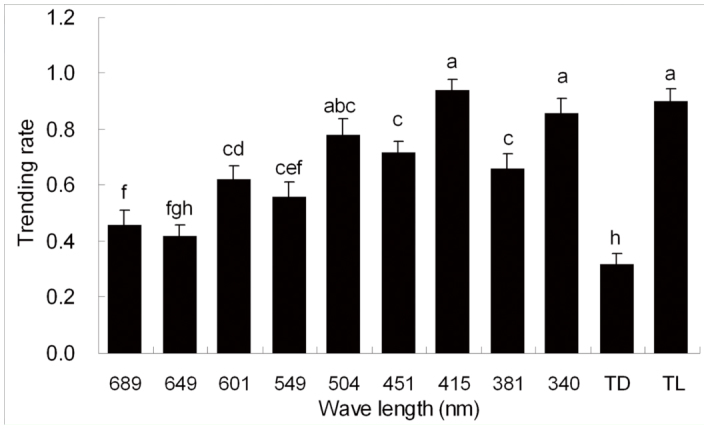
Mean (± SE) trending rates of *Pissodes punctatus* to monochromatic light of nine wavelengths, total darkness (TD), and total light (TL). Different letters on top of each bar indicate significant differences from one another by LSD (*p* < 0.05) after significant effect found by ANOVA. High quality figures are available online.

The differences in trending rates among total darkness, total light, and nine monochromatic lights were significant (ANOVA, F10,44 = 19.193, *p* < 0.001; Figure 2). Furthermore, the difference in trending rates between total darkness and total light was significant (LSD, *p* < 0.001; Figure 2). In total darkness, most *P. punctatus* stayed in the middle of the bioassay tube as non-responders (68.0 ± 3.7%); some stayed still, and others crawled around the inner wall of the bioassay tube. A few weevils (32.0 ± 3.7%) crawled from the releasing point towards one of the two ends of the bioassay tube for less than 20 cm. In total light, most *P. punctatus* (90.0 ± 4.5%) crawled towards one of the two ends of the bioassay tube, and all of the responsive weevils crawled more than 20 cm. A few weevils (10.0 ± 4.5%) stayed at the middle of bioassay tube as non-responders.

In the phototactic response experiments, significant differences were found in trending rates between total darkness and eight of nine monochromatic lights, the exception being red light (649 nm) (LSD, *p* < 0.05; Figure 2). The trending rate of the weevils under red light did not differ significantly from the trending rate in the total darkness treatment (LSD, *p* = 0.137; Figure 2). There were no significant differences in trending rates between the treatment under total light and those treatments under violet (415 nm), ultraviolet (340 nm), and green (504 nm) light (LSD, *p* > 0.05; Figure 2). The trending rates in response to violet and UV light were significantly higher than the trending rates in the six other monochromatic lights, including 689 nm, 649 nm, 601 nm, 549 nm, 451 nm, and 381 nm (LSD,*p* < 0.05; Figure 2).

### Response to light intensity

**Figure 3.**
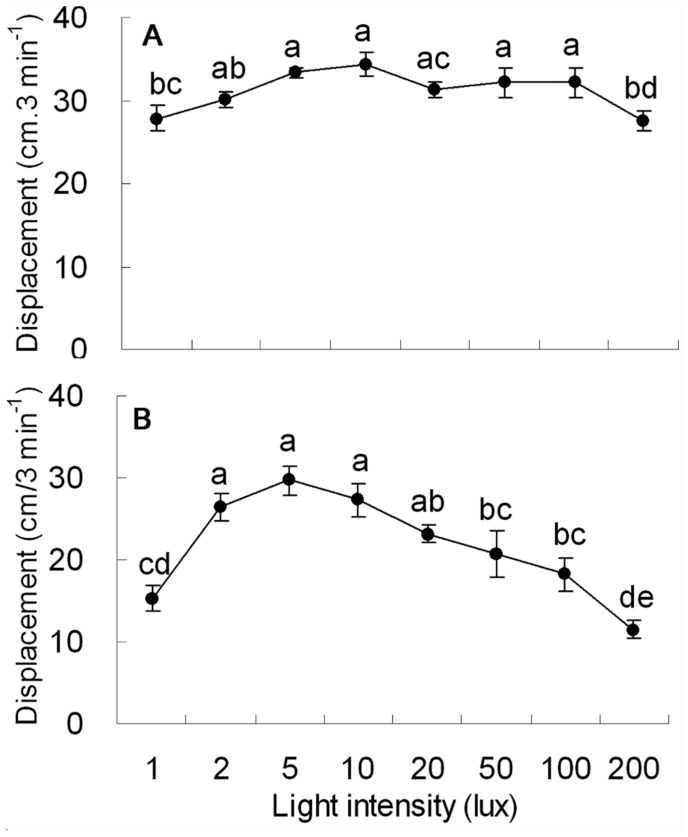
Mean (± SE) displacement of *Pissodes punctatus* adults to different intensities of violet light (41 5 nm, A) and ultraviolet light (340 nm, B). Different letters on top of each bar indicate significant differences from one another by LSD (*p* < 0.05) after significant effect found by ANOVA. High quality figures are available online.

The weevil stayed at the releasing point or crawled toward the light source when one end of the bioassay tube was illuminated by light at intensity above 1 lux (Figure 3). Significant differences in the displacement of *P. punctatus* were found among the eight intensities of violet light (ANOVA, F_7,32_ = 3.551, *p* < 0.01; Figure 3A). A significant difference in displacement was found between the treatment under a light intensity of 1 lux and the treatment under a light intensity of 5 lux (LSD, *p* < 0.01; Figure 3A). There was no significant difference in displacement among treatments of light intensity in 2 lux, 5 lux, 10 lux, 20 lux, 50 lux, and 100 lux (LSD, *p* >0.05; Figure 3A). A significant difference in displacement was found between treatments under a light intensity of 100 lux and 200 lux (LSD,*p* < 0.05; Figure 3A).

Significant differences in the displacement of *P. punctatus* were found among eight intensities of ultraviolet light (ANOVA, F_7,32_ = 11.760,*p* < 0.001; Figure 3B). A significant difference in displacement was found between the treatments under a light intensity of 1 lux and of 2 lux (LSD, *p* < 0.001; Figure 3B). There were no significant differences in displacement among treatments of light intensities of 2 lux, 5 lux, 10 lux, and 20 lux (LSD, *p* > 0.05; Figure 3B). A significant difference in displacement was found between treatments under light intensities of 10 lux and 50 lux (LSD, *p* < 0.05; Figure 3B) and between treatments under light intensities of 50 lux and 200 lux (LSD, *p* < 0.01; Figure 3B).

## Discussion

The results of the *P. punctatus* phototactic experiments suggested the following: (1) *P. punctatus* adults are diurnal and phototactic insects that show phototaxis to a broad spectrum of light and are most sensitive to violet, ultraviolet, and green light; (2) *P. punctatus* adults showed an increased phototactic response at low intensities, and a decreased phototactic response at high intensities for both violet and UV light.

*P. punctatus* adults were found to be diurnal and phototactic. The weevil was active in the daytime and became still at night or in a dark room. *P. punctatus* performed more tortuous walks in complete darkness, suggesting the orientation behavior of the weevil relies on light. Previous studies showed that other *Pissodes sp*. (Meng et al 2000; Wang 2009), such as *Tomicus piniperda* L. (Ye 2000), were phototactic insects. The phototaxis of most weevils suggests that solar energy is necessary for their movement (Ye 2000). In the present study, *P. punctatus* moved among host-trees by flying more than it did by crawling. Flying requires the use of more energy than crawling, so. *P. punctatus* might be more sensitive to light due to the importance of solar energy.

*P. punctatus* can identify a broad spectrum of monochromatic light, possibly being attracted to red (689 nm), orange (601 nm), green (549 nm, 504 nm), blue (451 nm), violet (415 nm), and ultraviolet light (381nm, 340 nm), while being blind to red (649 nm) light. Previous studies also showed that many beetles are attracted to a broad spectrum of light (Chen et al 2009; Ju et al 2010). Many insects inhabit a variety of habitats, with varied light environments caused by different geometry of light paths, different physical environment (Endler 1993; Bowmaker 1995), different daytime lengths and seasons (Thorne et al 2009), and different weather conditions. Thus, the capacity to identify a broad spectrum of light might help the insects adapt to varied light environments. In the present study, the sensitivity of *P. punctatus* to a broad spectrum of light would be helpful for its spread and migration among different host-trees in a variety of habitats.

Among a broad spectrum of attractive light, *P. punctatus* is most sensitive to the three monochromatic lights violet (415 nm), ultraviolet (340 nm), and green (504 nm). It is possible that there are at least three types of photoreceptors in the compound eye of *P. punctatus* (this species does not have any ocelli), and that this type of photoreceptor might drive its movement much more effectively than any other receptor types. This use of a photoreceptor to drive movement might be associated with a specialized capability of the beetle to use sky polarization in the UV region of the spectrum and/or the position of the sun as a course-stabilizing function during flights (Mishra and MeyerRochow 2006). Previous studies also showed that many beetles were most sensitive to shortwave lights such as violet and ultraviolet light (Yang et al. 2003; Guz et al. 2010; Ju et al 2010; Fernando and Joseph 2011). The sensitivity of *P. punctatus* to green light (504 nm) is also high. Previous studies also indicated that many beetles are phototactic to green light (Yang et al. 2003; Fernando and Joseph 2011). The results of the present study suggest that green light is a visual cue to find the green shoots of the Armand pine on which the weevil feed, given the high phototaxis of the weevil to green light.

The phototactic activity of *P. punctatus* is associated with light intensity. The displacement of *P. punctatus* increased at a low light intensity, kept stable within a special range, and decreased above that range for both ultraviolet and violet light. In many insects, the sign of phototaxis depends on the intensity of light so that low intensities elicit an increased phototactic response and high intensities a decreased phototactic response (Roberts et al 1992; Wei et al. 2000; Ye 2000; Liu et al. 2006; Wang et al. 2009). This switch above the special intensity of light allows the selection of optimum illumination, as unregulated phototaxis to the surface layers is dangerous because it exposes the organisms to damaging UV radiation (Jékely 2009). Phototaxis therefore has to be tightly controlled.

In conclusion, *P. punctatus* was most active in the day, was most phototactic to violet, UV, and green light, low intensities elicited an increased phototactic response, and high intensities a decreased phototactic response.. It has been shown that insects detect the canopy by their response to light (Bakke 1968; Ye 2000). Thus, *P. punctatus* might be trapped by utilizing the most attractive monochromatic light. Furthermore, *P. punctatus* might be controlled by increasing the density of the canopy of host plantations. Increasing the density would decrease the intensity of shorter wavelengths (Hamid 1995) and thereby reduce its attractiveness to the weevil.
